# Non-Gaussian Resistance Fluctuations in Gold-Nanoparticle-Based Gas Sensors: An Appraisal of Different Evaluation Techniques

**DOI:** 10.3390/s17040757

**Published:** 2017-04-03

**Authors:** Łukasz Lentka, Janusz Smulko, Mateusz Kotarski, Claes-Göran Granqvist, Radu Ionescu

**Affiliations:** 1 Faculty of Electronics, Telecommunications and Informatics, Gdańsk University of Technology, Narutowicza 11/12, 80-233 Gdańsk, Poland; lukasz.lentka@pg.gda.pl (Ł.L.); mateusz.kotarski@gmail.com (M.K.); 2 Department of Engineering Sciences, The Ångström Laboratory, Uppsala University, P.O. Box 534, SE-75121 Uppsala, Sweden; claes-goran.granqvist@angstrom.uu.se; 3 Rovira i Virgili University, ETSE-DEEEA, Department of Electronics, Carrer de l’Escorxador, 43003 Tarragona, Spain; radu.ionescu@urv.cat

**Keywords:** gas sensor, 1/*f* noise, gold nanoparticles, higher-order statistics, bispectrum, second spectrum, level-crossing statistics, Lévy-stable distribution

## Abstract

Volatile organic compounds, such as formaldehyde, can be used as biomarkers in human exhaled breath in order to non-invasively detect various diseases, and the same compounds are of much interest also in the context of environmental monitoring and protection. Here, we report on a recently-developed gas sensor, based on surface-functionalized gold nanoparticles, which is able to generate voltage noise with a distinctly non-Gaussian component upon exposure to formaldehyde with concentrations on the ppm level, whereas this component is absent, or at least much weaker, when the sensor is exposed to ethanol or to pure air. We survey four different statistical methods to elucidate a non-Gaussian component and assess their pros and cons with regard to efficient gas detection. Specifically, the non-Gaussian component was clearly exposed in analysis using level-crossing parameters, which require nothing but a modest computational effort and simple electronic circuitry, and analogous results could be reached through the bispectrum function, albeit with more intense computation. Useful information could be obtained also via the Lévy-stable distribution and, possibly, the second spectrum.

## 1. Introduction

Respiratory diseases may lead to changes in the human body’s ability to produce and process volatile organic compounds (VOCs), and exhaled breath analysis can be used for non-invasive lesion recognition [1,2,3]. Such analysis methods require cheap, fast, and reliable VOC detection during medical checkups of patients. Detection of VOCs is of much interest also for other applications, such as environmental monitoring and protection [4]. VOCs tend to bind to gold nanoparticles (AuNPs) [5], and chemical sensors based on ultra-pure monolayer-capped gold nanoparticles are good candidates for medical and other applications. These sensors can be relatively cheap and, importantly, are able to operate at room temperature. The pertinent nanomaterials can be fabricated via a two-step technique wherein AuNPs are first deposited onto a substrate by advanced gas deposition and these nanoparticles are, subsequently, surface-functionalized with organic ligands via a dip-coating process. The gas deposition equipment, and detailed procedures for nanoparticle fabrication and functionalization, has been reported elsewhere [6,7,8].

In this paper we present a detailed investigation of formaldehyde detection by fluctuation-enhanced sensing (FES) applied to AuNP-based gas sensors. This method utilizes low-frequency 1/*f*-like noise (flicker noise) as a source of information about the sensor’s ambient atmosphere and was proposed several years ago [9,10,11]. The noise originates from adsorption–desorption processes related to electrical charge transfer [12]. The measurement setup was presented in detail in an earlier paper of ours [8].

Analyses of stochastic signals, such as those inherent in FES, are usually limited to estimation of their power spectral density (PSD), denoted *S*(*f*), or of the slope of the product *f* ∙ *S*(*f*) [13,14,15]. These functions secure information about the intensity of the random signals and can be used for gas detection or prediction of gas concentration, as reported elsewhere [9,10], but further improvement of gas detection requires more advanced signal processing. Such an enhanced analysis can benefit from the fact that the sensors’ noise can display an unambiguous non-Gaussian component, whose presence opens several possibilities for refinement. The present study, which is exploratory in character, considers four options: (i) the bispectrum function, which is non-zero for a non-Gaussian component and extracts that component from the ubiquitous Gaussian noise [16,17]; (ii) level-crossing statistics, which can be readily obtained using an electronic setup, which is cheap and satisfies conditions of low-energy operation [18]; (iii) determination of the second spectrum, which requires computational complexity similar to that for extracting PSDs [19,20] and obtains information about any changes of the power spectra; and (iv) parameters of the Lévy-stable distribution [21], which may be easily estimated and, therefore, can be attractive for representing non-Gaussian random signals.

Random signals are completely characterized by their PSDs and probability distributions [13]. The assessment of a probability distribution for noise, and determination of any eventual deviation from a normal distribution within a range of a few standard deviations σ (e.g., ±3σ), requires recording of a few millions of noise samples and is much more time-consuming than estimation of *S*(*f*). When an extended recording time cannot be accepted for determining the probability density, one can evaluate other functions containing non-Gaussian components as elaborated in our present study.

## 2. Experimental Set-Up and Results

**Figure 1:**
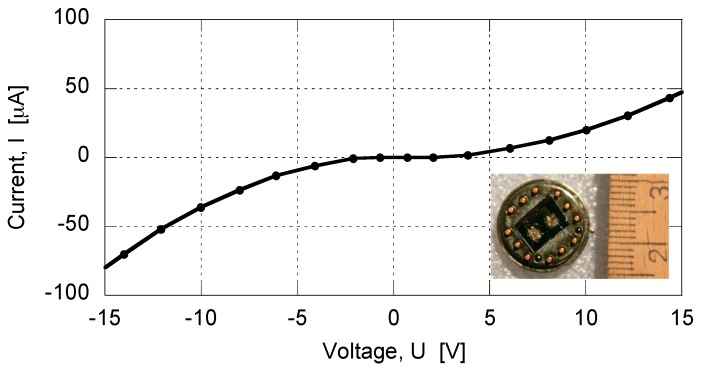
Current–voltage characteristic of an AuNP-based gas sensor in synthetic air. The inset depicts the sensor.

AuNP-based gas sensors were placed in a one-liter gas chamber and were exposed to either pure synthetic air (SA, 80% N_2_ and 20% O_2_) or to a target gas diluted with SA. The gas flow was ~0.05 liter/minute and was low enough to avoid turbulence. All measurements were performed at room temperature (22 °C). The sensors’ resistance lay between a few kΩ and a few MΩ. Initial electrical characterization was carried out by recording current–voltage (*I*–*U*) data, and Figure 1 displays a typical non-linear *I–U* curve with some asymmetry related to a Schottky junction formed between the gold electrodes and the gas-sensing material, which is a *p*-type semiconductor [8].

Detailed data acquisition used a system that simultaneously recorded a DC voltage and its fluctuations across the sensor by use of a precise DAQ card (NI-PCI 4474, National Instruments, Austin, TX, USA, characterized by 24-bit resolution ADCs with a dynamic range of 110 dB [22]) without amplification. About 8.2 × 10^6^ data samples were taken at a sampling frequency of 100 kHz for each type of gas. The use of such a vast number of samples reduced the random error of the estimated functions to about 1%. The recorded voltage samples are proportional to the sensor’s resistance fluctuations and are independent of the measurement setup [23].

The experiments were conducted as follows: the sensor was first exposed to pure SA for ~20 min after which the gas mixture was introduced during another 20 min, and voltage fluctuations were then recorded. The sensor’s bias voltage (*U_B_*) was set to either of two values, 5 or 11.3 V, during the measurements. A detailed description of the experiments can be found elsewhere [8].

No significant change in the sensors’ DC resistance could be detected upon exposure to pure SA or to a mixture of SA and a target gas comprised of 50 ppm of ethanol (C_2_H_5_OH) or 1.5 ppm of formaldehyde (HCHO), irrespective of the bias voltage.

**Figure 2:**
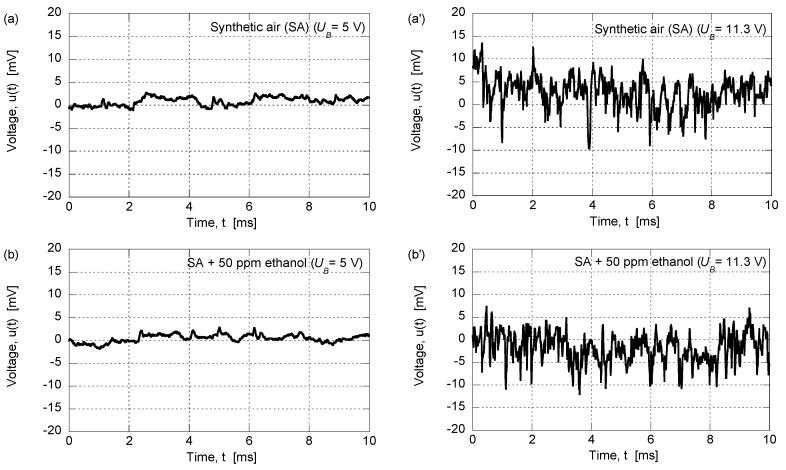

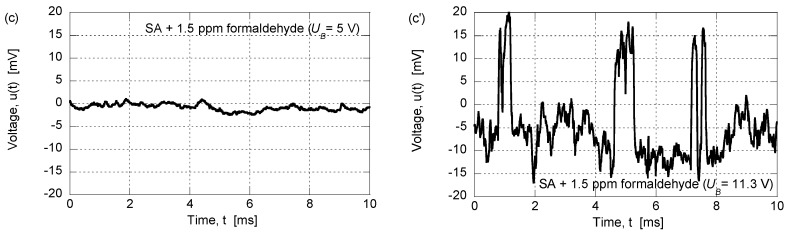
Time-dependent voltage fluctuations *u*(*t*) across an AuNP-based gas sensor exposed to the shown gases at the stated bias voltages (*U_B_*).

Fluctuating voltages were recorded across biased gas sensors, and Figure 2 reports data for (*i*) pure SA; (*ii*) SA with 50 ppm of ethanol; and (*iii*) SA with 1.5 ppm of formaldehyde, and with two magnitudes of *U_B_*. Recordings for *U_B_* = 5 V (left-hand panels) gave essentially Gaussian voltage fluctuations with an amplitude of about ±2 mV, irrespective of gas. At *U_B_* = 11.3 V, the amplitude of the Gaussian-type fluctuations was of the order of ±10 mV for SA, as well as for SA with 50 ppm of ethanol (upper and middle right-hand panels), whereas measurements for SA with 1.5 ppm of formaldehyde (lower right-hand panel) were qualitatively different and displayed a train of intense short spike-pulses with amplitudes of roughly ±20 mV. Our previous work [8] demonstrated that a histogram of the spike-like voltage fluctuations was consistent with the presence of a random telegraph signal (burst noise), which is in line with data for other semiconductor devices [24,25,26,27,28,29,30].

**Figure 3:**
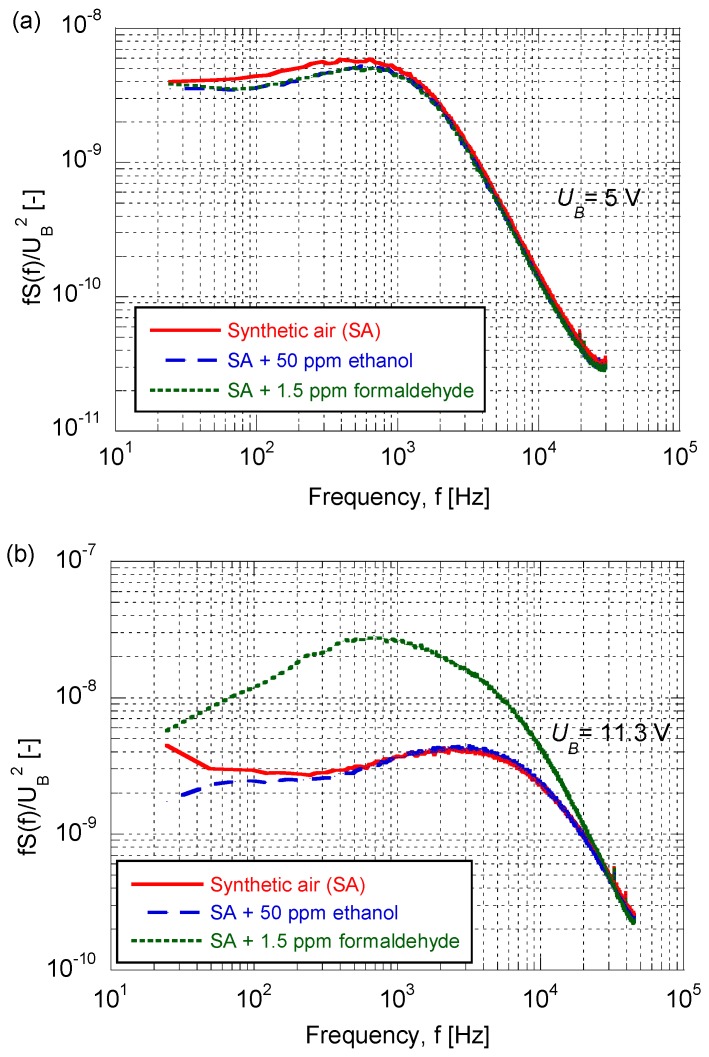
Power spectral density *S*(*f*) of an AuNP-based sensor’s voltage fluctuations, multiplied by frequency *f* and normalized by the square of the sensor’s bias voltage *U_B_*, upon exposure to the shown gases.

PSDs of the voltage fluctuations were determined for each of the three gases, as illustrated in Figure 3. It is evident that the data for SA and for SA mixed with 50 ppm of ethanol are almost indistinguishable for both bias voltages, whereas striking differences in the PSDs can be noted when the sensor was exposed to SA containing formaldehyde. The difference among the PSDs is most pronounced for the high bias voltage and then amounts to a factor of approximately ten at a frequency of 1 kHz; the effect is related to the spike-pulses noted in Figure 2c’.

## 3. Non-Gaussian Measures and Discussion

PSD data clearly illustrated the presence of formaldehyde when a sufficiently high bias voltage was applied to the AuNP-based gas sensor. However, it is reasonable to suppose that the characteristic short spike-pulses can be exposed and quantified even more clearly through noise parameters that highlight non-Gaussianity. In order to explore this possibility, we define a number of cumulants pertaining to a random discrete signal *x*(*n*), specifically the second [*C*_2*x*_(*k*)], third [*C*_3*x*_(*k*,*l*)], and fourth [*C*_4*x*_(*k*,*l*,*m*)] cumulants defined, respectively, by:

(1) $$C_{2x}\left(k\right)=E\left\{x\left(n\right)\cdot x\left(n+k\right)\right\},$$

(2) $$C_{3x}\left(k,l\right)=E\left\{x\left(n\right)\cdot x\left(n+k\right)\cdot x\left(n+l\right)\right\},$$

(3) $$C_{4x}\left(k,l,m\right)=E\left\{x\left(n\right)\cdot x\left(n+k\right)\cdot x\left(n+l\right)\cdot x\left(n+m\right)\right\},$$

where *E*{…} denotes averaging and *n*, *k*, *l*, and *m* are 0, 1, 2, 3, …. Cumulants calculated for zero lag (i.e., for *k* = *l* = *m* = 0) have special designations: *C*_2*x*_(0) is variance $\sigma_{x}^{2}$, *C*_3*x*_(0, 0)/$\sigma_{x}^{3}$ is skewness, and *C*_4*x*_(0, 0, 0)/$\sigma_{x}^{4}$ is kurtosis. Skewness signifies the symmetry of a probability distribution, whereas kurtosis is a measure of the relative “peakedness”. Both skewness and kurtosis are equal to zero if *x*(*n*) has a Gaussian distribution [31].

Skewness and kurtosis were evaluated for voltage fluctuations recorded across AuNP-based gas sensors. Such data are reported in Table 1 from which it is manifest that the fluctuations indeed show unambiguous evidence for non-Gaussian performance. Furthermore, the skewness and kurtosis were altered when formaldehyde was present. Interestingly, the relative change of the kurtosis was greatest at the low bias voltage, whereas the relative change of the skewness was greatest at the high voltage.

### 3.1. Bispectrum Function

The bispectrum is a second-order Fourier transform of the third-order cumulant (Equation (2)) and is a function of two frequencies, *f*_1_ and *f*_2_, according to:

(4) $$S_{3x}\left(f_{1},f_{2}\right)=\sum_{k=-\infty }^{\infty }\sum_{l=-\infty }^{\infty }C_{3x}\left(k,l\right)e^{-j2\pi f_{1}k}e^{-j2\pi f_{2}l}.$$

This function is either zero or a constant for Gaussian processes. The bispectrum function of a signal that is the sum of two statistically-independent processes is equal to the sum of the individual bispectra [16] and, hence, the higher-order cumulants of a non-Gaussian signal can be recovered even in the presence of Gaussian noise. The bispectrum displays axial symmetries for stationary random processes.

**Figure 4:**
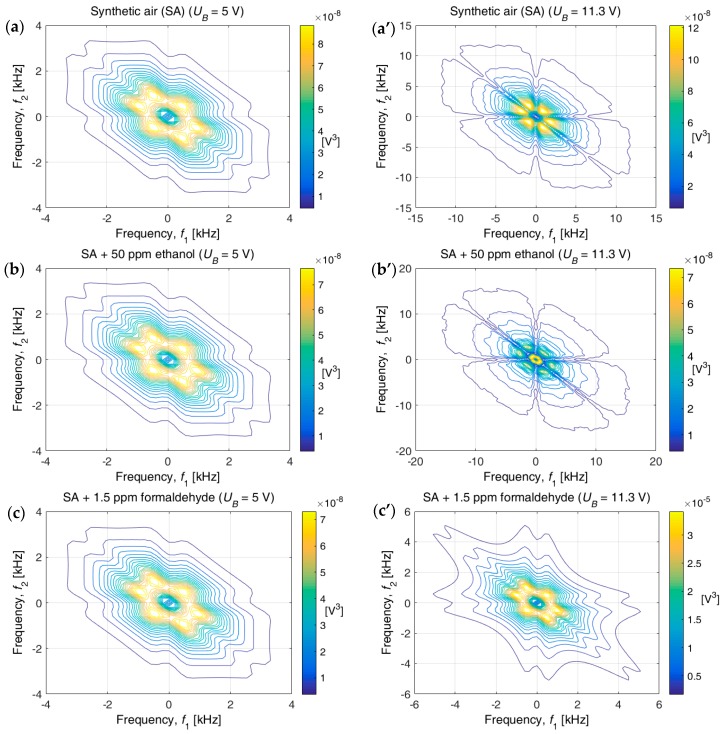
Two-dimensional contour plots of the bispectrum function for voltage fluctuations recorded across an AuNP-based gas sensor exposed to the shown gases at the stated bias voltages (*U_B_*). In each panel, the curves indicate equally-spaced color-coded constant values of the bispectrum function; they represent magnitudes ranging from a low limit (blue) to a high limit (yellow). Actual values of these limits are indicated by the colored vertical bars in the respective panels.

Figure 4 shows contour plots of bispectrum functions estimated for voltage fluctuations recorded across a AuNP-based sensor exposed to the same three gases as above at *U_B_* = 5 V (left-hand panels) and *U_B_* = 11.3 V (right-hand panels). The bispectrum functions were estimated by use of the BISPECD function available in MATLAB’S Higher-Order Spectral Analysis Toolbox (HOSA) [32]. A set of 256 samples, and 50% overlapping, were applied to obtain the data.

The shapes of the bispectrum functions look virtually the same for the three gases at *U_B_* = 5 V, whereas the differences among the gases are striking at *U_B_* = 11.3 V. It is, hence, evident that the bispectrum function offers a convenient pictorial method for gas discrimination. Different frequency ranges were used to display the data in the various panels of Figure 4 with the object of creating easily comparable images; the selection of appropriate range can be done automatically by different algorithms.

### 3.2. Level-Crossing Statistics

Random signals can be characterized by statistics of their level-crossings at arbitrary levels, and the relevant parameters—such as the mean value or variance of the level-crossing statistics—can easily be determined and compared in order to detect differences in random signals [18]. Thus, one can analyze voltage fluctuations *u*(*i*) via a new random variable *z*(*i*) given by:

(5) $$z\left(i\right)=\{\begin{array}{c} 1,\;\mathrm{if}\;u\left(i\right)\geq m_{u}\\ 0,\;\mathrm{if}\;u\left(i\right)<m_{u} \end{array},$$

where *m_u_* is the mean value of the voltage-sample series *u*(*i*) (*i* = 1 … *N*), and it is then possible to obtain a signal *w*(*i*) according to:

(6) w(i)=|z(i)−z(i−1)| (i = 2…N).

When *w*(*i*) attains a value of one, this condition means that at the moment in case—corresponding to a sample number *i*—the signal *u*(*i*) has crossed its mean value *m_u_*. All elements of the time series *w*(*i*) must be added to obtain the total number of level crossings. Next, one can establish statistic parameters such as the mean *m_w_* and variance $\sigma_{w}^{2}$ of the level-crossing events. Values of these parameters were evaluated for AuNP-based sensors; data are given in Table 2 and indicate whether different gases can be detected by level-crossing statistics.

It is evident that similar level-crossing parameters were obtained for the three gases in the case of *U_B_* = 5 V, and similar parameters were also found for pure SA and for a mixture of SA and 50 ppm of ethanol at *U_B_* = 11.3 V. However, the parameters were distinctly different—by as much as a factor ~5 for the variance—when the sensor was exposed to SA with 1.5 ppm of formaldehyde. These results prove that level-crossing statistics is able to secure decisive information for formaldehyde detection by AuNP-based gas sensors. It should be underscored that the level-crossing statistics can be acquired by an electronic circuit comprising a comparator and a register, i.e., by a cheap microcontroller.

### 3.3. Second Spectrum

Methods based on the collection of fourth moments are popular for analyzing random data containing non-Gaussian components, and the technique is related to what is known as a “second spectrum” $S_{2}\left(f_{2},f_{L},f_{H}\right)$ [19,20]. This function can be estimated by repeatedly measuring *S*(*f*) around each frequency within a range from a low value *f_L_* to a high value *f_H_*, and the ensuing series of PSDs can then be transformed into a new frequency domain *f*_2_ by use of a Fourier transform. The second spectrum is frequency-independent for Gaussian signals.

An appropriate procedure for determining the second spectrum was developed in MATLAB software, following a description in the literature [19]. The voltage fluctuations *u*(*t*) were divided into 64,000 non-overlapping samples *u_ss_*(*t*), each of a time length *T_ss_* = 1.28 × 10^−3^ s, and all *u_ss_*(*t*) samples were utilized to estimate the PSDs. Each power spectrum was averaged over a set of eight PSD estimators to reduce the random error. The estimated data on *S*(*f*) had a frequency resolution of Δ*f* = 1/(8*T_ss_*) ≈ 98 Hz. In the next step, each PSD was summed up within the frequency range from *f_L_* = Δ*fn_L_* ≈ 98 Hz to *f_H_* = Δ*fn_H_* ≈ 50 kHz, where *n_L_* and *n_H_* denote the number of the frequency bin for the lowest and highest frequency, respectively. These sums created a new data vector *P*(*t*) with a length of ~8000. That vector was divided into 15 non-overlapping segments, each of 512 samples, and was employed to derive the second spectrum by use of a Fourier transform and squaring. The frequency resolution of $S_{2}\left(f_{2},f_{L},f_{H}\right)$ is equal to Δ*f*_2_ = 1/(512 · *T_ss_*) ≈ 0.19 Hz. The second spectra were normalized by an average value of *P*(*t*).

**Figure 5:**
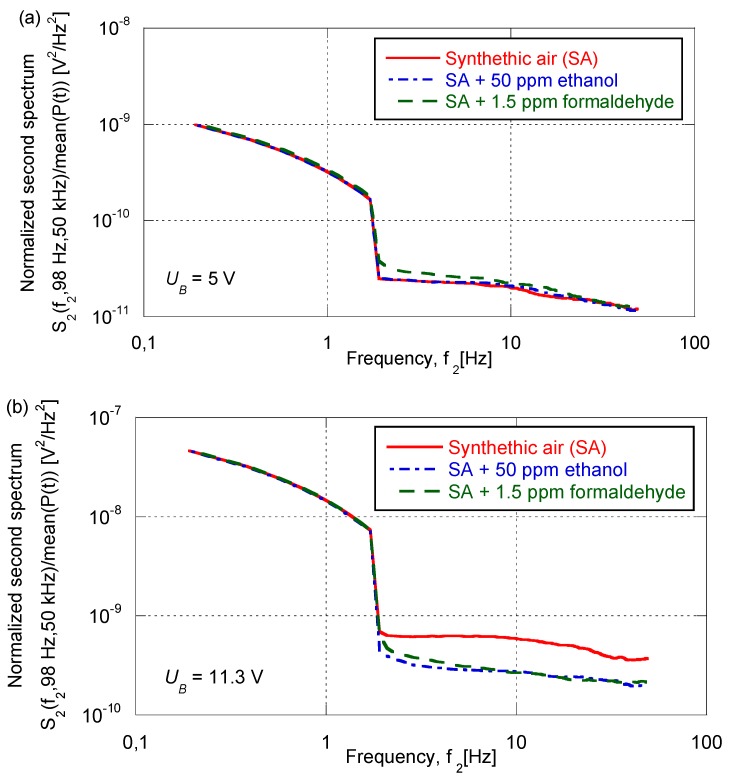
Second spectra for voltage fluctuations across an AuNP-based gas sensor exposed to the shown gases at the stated bias voltage (*U_B_*). The spectra were normalized to attain the same level at *f*_2_ = 0.19 Hz.

Figure 5 presents second spectra pertinent to voltage fluctuations across an AuNP-based sensor. The data for the various gases were similar at *U_B_* = 5 V (upper panel). However, a clear difference was noted in the case of pure SA for frequencies above ~2 Hz at *U_B_* =11.3 V (lower panel). The data are consistent with voltage fluctuations in the case of pure SA being more Gaussian-like than when ethanol or formaldehyde was present, which indicates that the second spectra are able to pinpoint features that are not captured by the other statistical analyses.

### 3.4. Lévy-Stable Distribution

The Lévy-stable distribution (LSD) comprises a wide range of probability distributions with strong (non-exponential) tails and skewness [21] and is, therefore, expected to be suitable for characterizing spike trains. Many studies have shown that LSD parameters can serve as a powerful tool for signal processing, with specific examples found in areas such as geology [33], human balance control [34], noise theory [34,35,36,37], etc. One particular investigation [33] demonstrated that the LSD can be employed for analysing nonstationary processes with stationary increments that can be treated like random motion instead of random noise. The same study [33] also emphasized that fractional Lévy motions displayed a high degree of variability and tended to undergo occasional large jumps—analogous to features of the voltage signal examined in the present experiments (*cf*. Figure 2c’).

In general terms, the LSD is defined by its characteristic function and is specified completely by four parameters: stability index *α* (0 < *α* ≤ 2), skewness parameter *β* (–1 ≤ *β* ≤ 1), scale parameter *γ* (>0), and location parameter *δ*. The well-known Gaussian and Cauchy distributions constitute special cases of the LSD when its stability index is equal to 2 and 1, respectively. Table 3 presents estimated values of the four LSD parameters based on noise voltage records observed with the AuNP-based gas sensor. Specifically, the evaluation used the *levystblfit* function in the MATLAB toolbox [38].

The LSD parameters for a bias voltage of 5 V did not differ strongly irrespectively of which gas was used. However, the skewness parameter was distinctly different for *U_B_* = 11.3 V when formaldehyde was present, which signifies that this gas induced a non-Gaussian noise component (specifically, as shown above, a train of voltage spikes). On the other hand, the scale parameter and the location parameter remained rather stable when the gas was changed. The results in Table 3 prove convincingly that the Lévy-stable distribution can be used to detect formaldehyde.

## 4. Conclusions

The present investigation reported analytic results for stochastic signals generated in a prototype resistive gas sensor based on monolayer-capped gold nanoparticles. Specifically, we applied several statistical methods for extracting a non-Gaussian component from 1/*f*-like voltage noise. This component could be very intense when the sensor’s ambient atmosphere contained formaldehyde with a concentration as small as on the ppm level. The relative changes of some of the statistical parameters or functions were large, which opens new avenues towards accurate gas detection, and formaldehyde could be probed by at least some of the methods. The *bispectrum method* function yielded distinct information, provided that the bias voltage across the sensor was large enough, and gas discrimination could be represented pictorially; this method relies on intense computation. *Level-crossing analysis* is another option for formaldehyde detection and is noteworthy since it can be performed with nothing but elementary arithmetic operations and implemented by use of conventional electronic circuitry. Parameters of the *Lévy-stable distribution* present still another possibility and, in particular, skewness parameters were sensitive to the presence of formaldehyde if the bias voltage was sufficiently large. Finally, the *second spectrum* can yield gas-specific information, but its interpretation needs more in-depth analysis.

With regard to applications, our results demonstrate how the sensitivity of a novel sensor can be boosted via statistical analyses focused on non-Gaussian signals.

## Figures and Tables

**Table 1:** Dimensionless kurtosis and skewness of voltage fluctuations across an AuNP-based gas sensor exposed to the shown gases at the stated bias voltages (*U_B_*).

	*U_B_* = 5 V			*U_B_* = 11.3 V		
	Synthetic Air (SA)	SA + 50 ppm Ethanol	SA + 1.5 ppm Formaldehyde	SA	SA + 50 ppm Ethanol	SA + 1.5 ppm Formaldehyde
Kurtosis [-]	3.961	4.031	4.465	3.582	3.639	3.642
Skewness [-]	0.589	0.592	0.518	−0.133	−0.116	0.961

**Table 2:** Level-crossing parameters for voltage fluctuations at a time rate *T_lc_* equal to 10^−5^ s across an AuNP-based gas sensor for the shown gases and bias voltages (*U_B_*).

	*U_B_* = 5 V			*U_B_* = 11.3 V		
	Synthetic Air (SA)	SA + 50 ppm Ethanol	SA + 1.5 ppm Formaldehyde	SA	SA + 50 ppm Ethanol	SA + 1.5 ppm Formaldehyde
$\overset{N}{\underset{i=2}{\sum }}w\left(i\right)$ [-]	357,740	387,567	384,793	777,278	814,295	409,993
$m_{w}\cdot T_{lc}\;[\mathrm{ms}]$	0.229	0.211	0.213	0.105	0.101	0.200
$\sigma_{w}^{2}\cdot T_{lc}^{2}$ [s^2^]	3.35 × 10^−7^	3.06 × 10^−7^	3.33 × 10^−7^	3.60 × 10^−8^	2.76 × 10^−8^	1.42 × 10^−7^

**Table 3:** Dimensionless stability index (*α*), skewness parameter (*β*), scale parameter (*γ*), and location parameter (*δ*) characterizing Lévy-stable distributions for voltage fluctuations across an AuNP-based gas sensor for the shown gases and bias voltages (*U_B_*).

	*U_B_* = 5 V			*U_B_* = 11.3 V		
	Synthetic air (SA)	SA + 50 ppm Ethanol	SA + 1.5 ppm Formaldehyde	SA	SA + 50 ppm Ethanol	SA + 1.5 ppm Formaldehyde
*α* [-]	1.9699	1.9730	1.9727	1.6903	1.6999	1.4640
*β* [-]	0.7315	0.7101	0.7281	−0.3609	−0.3459	−0.0510
*γ* [-]	0.0001	0.0001	0.0001	0.0008	0.0009	0.0008
*δ* [-]	9.297 × 10^−7^	7.959 × 10^−7^	8.287 × 10^−7^	−3.376 × 10^−5^	−3.35 × 10^−5^	−2.582 × 10^−5^

## References

[1] Mazzone P.J. (2010). Exhaled volatile organic compounds as biomarkers for respiratory diseases. Eur. Respir. Monogr..

[2] Peng G., Tisch U., Adams O., Hakim M., Shehada N., Broza Y.Y., Billan S., Abdah-Bortnyak R., Kuten A., Haick H. (2009). Diagnosing lung cancer in exhaled breath using gold nanoparticles. Nat. Nanotechnol..

[3] Xu Z.-Q., Broza Y.Y., Ionsecu R., Tisch U., Ding L., Liu H., Song Q., Pan Y.-Y., Xiong F.-X., Gu K.-S. (2013). A nanomaterial-based breath test for distinguishing gastric cancer from benign gastric conditions. Br. J. Cancer.

[4] Ho C.K., Robinson A., Miller D.R., Davis M.J. (2005). Overview of sensors and needs for environmental monitoring. Sensors.

[5] Grönbeck H., Curioni A., Andreoni W. (2000). Thiols and disulfides on the Au (111) surface: The headgroup-gold interaction. J. Am. Chem. Soc..

[6] Granqvist C.G., Buhrman R.A. (1976). Ultrafine metal particles. J. Appl. Phys..

[7] Ederth J., Kish L.B., Olsson E., Granqvist C.G. (2002). In situ electrical transport during isothermal annealing of nanocrystalline gold films. J. Appl. Phys..

[8] Lentka Ł., Kotarski M., Smulko J., Cindemir U., Topalian Z., Granqvist C.G., Calavia R., Ionescu R. (2016). Fluctuation-enhanced sensing with organically functionalized gold nanoparticle gas sensors targeting biomedical applications. Talanta.

[9] Kish L.B., Vajtai R., Granqvist C.G. (2000). Extracting information from noise spectra of chemical sensors: Single sensor electronic noses and tongues. Sens. Actuators B Chem..

[10] Kotarski M.M., Smulko J.M. (2010). Hazardous gases detection by fluctuation-enhanced gas sensing. Fluctuation Noise Lett..

[11] Kotarski M., Smulko J., Czyżewski A., Melkonyan S. (2011). Fluctuation-enhanced scent sensing using a single gas sensor. Sens. Actuators B Chem..

[12] Weisz P.B. (1953). Effect of electronic charge transfer between adsorbate and solid on chemisorption and catalysis. J. Chem. Phys..

[13] Bendat J.S., Piersol A.G. (2011). Random Data: Analysis and Measurement Procedures.

[14] Kiwilszo M., Smulko J. (2009). Pitting corrosion characterization by electrochemical noise measurements on asymmetric electrodes. J. Solid State Electrochem..

[15] Schmera G., Kish L.B. (2002). Fluctuation enhanced chemical sensing by surface acoustic wave devices. Fluct. Noise Lett..

[16] Mendel J.M. (1991). Tutorial on higher-order statistics (spectra) in signal processing and system theory: Theoretical results and some applications. Proc. IEEE.

[17] Smulko J., Granqvist C.G., Kish L.B. (2001). On the statistical analysis of noise in chemical sensors and its application for sensing. Fluct. Noise Lett..

[18] Gingl Z., Kish L.B., Ayhan B., Kwan C., Granqvist C.G. (2010). Fluctuation-enhanced sensing with zero-crossing analysis for high-speed and low-power applications. IEEE Sens. J..

[19] Kolek A. Digital estimation of second spectra. Proceedings of the 23rd International Conference on Noise and Fluctuations.

[20] O’Brien K.P., Weissman M.B. (1994). Statistical characterization of Barkhausen noise. Phys. Rev. E.

[21] Lévy P. (1925). Calcul des Probabilités.

[22] NI PCI-4474. http://sine.ni.com/nips/cds/view/p/lang/pl/nid/12205.

[23] Kotarski M., Smulko J. (2009). Noise measurement setups for fluctuations enhanced gas sensing. Metrol. Meas. Syst..

[24] Voss R.F. (1978). Linearity of 1/*f* noise mechanisms. Phys. Rev. Lett..

[25] Yakimov A.V., Hooge F.N. (2000). A simple test of the Gaussian character of noise. Physica B.

[26] Hung K.K., Ko P.K., Ho C., Cheng Y.C. (1990). Random telegraph noise of deep-submicrometer MOSFET’s. IEEE Electr. Device Lett..

[27] Pavelka J., Šikula J., Tacano M., Toita M. (2011). Activation energy of RTS noise. Radioengineering.

[28] Raoult J., Militaru L., Verdier J., Souifi A. (2002). Time domain and frequency analysis of RTS noise in deep submicron SiGe HBTs. Nucl. Instrum. Meth. Phys. Res. B.

[29] Crook R., Jones B.K. (1997). Noise and DC characteristics of power silicon diodes. Microelectron. Reliab..

[30] Konczakowska A., Cichosz J., Szewczyk A. (2006). A new method for identification of RTS noise. Bull. Pol. Acad. Sci. Tech. Sci..

[31] Hinich M.J. (1982). Testing for Gaussianity and linearity of a stationary time series. J. Time Ser. Anal..

[32] Swami A., Mendel J.R., Nikias C.L. (1993). Higher-Order Spectral Analysis Toolbox User’s Guide.

[33] Painter S. (1995). Random fractal models of heterogeneity: The Lévy-stable approach. Math. Geol..

[34] Cabrera J.L., Bormann R., Eurich C., Ohira T., Milton J. (2004). State-dependent noise and human balance control. Fluct. Noise Lett..

[35] Chechkin A.V., Sliusarenko O.Yu., Metzler R., Klafter J. (2007). Barrier crossing driven by Lévy noise: Universality and the role of noise intensity. Phys. Rev. E.

[36] Montroll E.W., Shlesinger M.F. (1982). On 1/*f* noise and other distributions with long tails. Proc. Natl. Acad. Sci. USA.

[37] Eliazar I., Klafter J. (2009). A unified and universal explanation for Lévy laws and 1/*f* noises. Proc. Natl. Acad. Sci. USA.

[38] Liang Y., Chen W. (2013). A survey on computing Lévy stable distributions and a new MATLAB toolbox. Signal Process..

